# Beyond Stress Granules: G3BP1 and G3BP2 Redundantly Suppress SARS-CoV-2 Infection

**DOI:** 10.3390/v17070912

**Published:** 2025-06-27

**Authors:** Duo Xu, Mahamaya Biswal, Quanqing Zhang, Christine Light, Yijie Wu, Chenjin Ye, Luis Martínez-Sobrido, Jikui Song, Rong Hai

**Affiliations:** 1 Department of Microbiology and Plant Pathology, University of California-Riverside, Riverside, CA 92521, USA; duox@ucr.edu (D.X.); christine.light@email.ucr.edu (C.L.); ywu548@ucr.edu (Y.W.); 2 Department of Biochemistry, University of California-Riverside, Riverside, CA 92521, USA; mbisw002@ucr.edu (M.B.); jikuis@ucr.edu (J.S.); 3 Institute for Integrative Genome Biology, Proteomics Core, University of California-Riverside, Riverside, CA 92521, USA; quanqinz@ucr.edu; 4 Texas Biomedical Research Institute, San Antonio, TX 78227, USA; chenjinye@txbiomed.org (C.Y.); lmartinez@txbiomed.org (L.M.-S.)

**Keywords:** SARS-CoV-2, virus–host interplay, stress granule, virulence, virus replication

## Abstract

The global pandemic caused by severe acute respiratory syndrome coronavirus 2 (SARS-CoV-2) has posed unprecedented challenges to public health and economic stability. Central to SARS-CoV-2 pathogenesis is its ability to evade the host immune response by hijacking host pathways via the interaction between viral and host proteins. We identified Ras-GTPase-activating protein SH3 domain-binding protein 1/2 (G3BP1/G3BP2) as a critical host factor that interacts with the viral nucleocapsid (N) protein, emerging from a comparative analysis of proteomic data from multiple studies. We revisited the underlying molecular mechanisms by confirming the residues required for the interaction between G3BP1/G3BP2 and SARS-CoV-2 N protein and showed that this interaction disrupts stress granule formation. Intriguingly, we observed that the ablation of both G3BP1 and G3BP2 enhanced SARS-CoV-2 replication. Our data collectively supports the notion that G3BP1 and G3BP2 play a critical role in modulating the host–virus interface during SARS-CoV-2 infection, and that their multifaceted function in cellular defense extends beyond the stress granule pathway.

## 1. Introduction

Severe acute respiratory syndrome coronavirus 2 (SARS-CoV-2), the etiologic agent of coronavirus disease 2019 (COVID-19), has exacted a substantial toll on global health and economic stability [1]. For SARS-CoV-2 to establish a productive infection, it must effectively evade the host immune responses. Recent investigations have shown that the virus employs a repertoire of viral proteins to actively circumvent immune detection [2]. One such protein is the SARS-CoV-2 nucleocapsid (N) protein, which comprises two RNA-binding domains and plays a pivotal role in modulating the interferon (IFN) signaling pathway [3,4,5]. Crystallographic analysis of the N protein-RNA complex (PDB: 7ACT) confirmed that SARS-CoV-2 N protein also encapsulates the viral RNA genome and facilitates its interaction with the viral envelope [5,6]. Notably, the N protein also plays a crucial role in modulating host pathways to facilitate SARS-CoV-2 infection. For instance, the SARS-CoV-2 N protein appears to subvert the IFN-induced signaling cascade by inhibiting the phosphorylation of STAT1 and STAT2 transcription factors [3], and may also impinge upon additional components of the pathway (e.g., stress granule pathway) to evade the host’s antiviral response [7,8,9,10,11,12,13,14,15,16,17,18,19,20,21,22]. Despite the critical role of the N protein in the viral life cycle and its association with numerous host factors, the key host factors involved, as well as the molecular determinants governing these interactions, remain unknown.

The advent of genome-scale RNA interference (RNAi) screening and global affinity purification–mass spectrometry (AP-MS) approaches has revolutionized our understanding of host–pathogen interactions, enabling the elucidation of complex molecular relationships at an unprecedented level of resolution. To date, numerous studies have used global AP-MS approaches to elucidate the interactome of SARS-CoV-2 N protein, yielding a wealth of information on its molecular interactions [23,24,25,26]. Despite the merits of systems-level approaches, they are inevitably encumbered by the twin pitfalls of false positives and false negatives. To mitigate these inherent errors, we proposed that the overlaps of host proteins identified across multiple studies would serve as a filtering mechanism, thereby refining our analysis and enhancing confidence in the authenticity of protein–protein interactions involving SARS-CoV-2 N protein. By intersecting these datasets, we were able to distill the essence of SARS-CoV-2 N protein, revealing the true hub factors that orchestrate its replication and pathogenesis. The observed congruity among these datasets will significantly enhance our ability to leverage these important findings to gain further mechanistic insights into the role of specific host proteins in the SARS-CoV-2 replicative cycle, ultimately informing the development of host-directed therapeutic strategies.

Herein, we have been able to identify key host cellular factors that are associated with SARS-CoV-2 N protein. To determine the hub protein associated with viral N protein, we performed a comparative analysis of our affinity AP-MS datasets with those generated by previous studies [23,24,25,26]. Notably, Ras-GTPase-activating protein SH3 domain-binding proteins 1 and 2 (G3BP1 and 2) emerged as the sole host factors that were consistently detected across all datasets. Furthermore, our findings revealed that the absence of both G3BP1 and G3BP2 proteins correlated with an enhanced replication of SARS-CoV-2, thereby underscoring the likely overlapping antiviral function of these cellular proteins in the context of SARS-CoV-2 infection. We subsequently explored the molecular mechanisms underlying the interaction between G3BP1/2 and the N protein, as well as the role of this interaction in the formation of stress granules (SGs). Our study provides novel insights into the molecular mechanisms that govern SARS-CoV-2 replication and highlights the potential of targeting the G3BP1/2-N protein interaction as a promising host-directed therapeutic strategy for the treatment of SARS-CoV-2 infection.

## 2. Materials and Methods

### 2.1. Cell Lines and Viruses

E6-Vero (African green monkey kidney epithelial cells), 293T, and A549 cells, with hACE2 expression, were purchased from ATCC and cultured in high-glucose DMEM supplemented with 5 mM L-glutamine and 10% FBS. All cells were tested as mycoplasma-negative using the PlasmoTest kit (InvivoGen, Hong Kong), following the standard protocol. The sub-cultures were validated by morphological evaluation under a light microscope regularly. SARS-CoV-2 (USA-WA1/2020) viruses, kindly provided by Dr. Luis Martinez-Sobrido at the Texas Biomedical Research Institute, were grown in E6-Vero cells. Virus titers were determined by plaque assays with E6-Vero cells.

### 2.2. Growth Curve of SARS-CoV-2 and Plaque Assays

To establish the growth curves of SARS-CoV-2, A549 and G3BP KO cells were infected with SARS-CoV-2 (USA-WA1/2020) virus in a 12-well plate (MOI:1.0). The virus was allowed to infect cells for 1 h, then the media were changed to 1mL of post-infection media (DMEM containing 1% Penn/Strep, 2% FBS). Then, 50 µL of media was collected at 6, 9, and 12 h post-infection. The virus titer at each time point post-infection was determined by plaque assays. The collected viruses were serially diluted 10-fold in PBS. Then, 200 µL of virus dilution was added to a confluent monolayer of Vero cells in a 12-well plate, and the virus was allowed to infect cells for 1 h at 37 °C. Virus dilutions were replaced with Plaquing Media (post-infection media containing 0.6% Avicell) and left at 37 °C for 72 h. Cells were fixed with 1 mL of 3.7% paraformaldehyde for 1 h at room temperature and stained with 1% crystal violet solution.

### 2.3. Generation of G3BP-Knockout Cell Lines by CRISPR-Cas9

To generate G3BP1-knockout cell lines, the guide RNAs (5′-GCTCATGCCACGCTAAATGA-3′, and 5′-AACGTTTGTCCTTGCTCCTG-3′) were designed to target exon 3 of the G3BP1 gene and were cloned into the PX459 vector (Addgene, Watertown, MA, USA). The plasmids were co-transfected into A549 cells. At 48 h post-transfection, transfected cells were selected with puromycin (3 μg/mL) for 48 h. Single clones of G3BP1-knockout cells were isolated and expanded in 24-well plates for further screening. We confirmed the knockout status of these cells through final sequencing analysis. For G3BP2-knockout cell lines, we used a similar approach, with guide RNAs (5′-AAGCTCCGGAATATTTACAC-3′ and 5′-CTGCTTGTAGGGCGGGAGTT-3′) targeting exon 1 of the G3BP2 gene. For G3BP double-knockout (DKO) cell lines, we used a similar approach and the same two sets of guide RNAs used earlier.

### 2.4. Plasmids and Immunoprecipitation

The DNA fragment encoding SARS-CoV-2 N (1260 bp) was synthesized from Thermo Fisher Scientific. The cDNAs for full-length hG3BP1 and hG3BP2 were purchased from the DNASU plasmid repository. The full-length SARS-CoV-2 N was cloned in a PCAGGS-C-FLAG vector, and the full-length G3BP1 and G3BP2 were cloned into a pCAGGS-N-myc vector, for which expression was under the control of the chicken β actin promoter.

Then, 8 × 10^6^ 293T cells were plated in five l50 cm^2^ tissue culture dishes the day before transfection. They were transfected with 30 µg of SARS-CoV-2 N-FLAG using polyethylenimine (PEI). At 48 h post-transfection, cells were washed in 1× PBS twice and collected for lysis in NP-40 buffer (0.5% NP-40, 50 mM Tris (pH 8.0), 100 mM NaCl, 10 mM MgCl_2_, 10% glycerol, and 1 mM DTT) supplemented with 1 mM PMSF. The lysis procedure was performed by rotation end-over-end at 4 °C for 20 min, followed by centrifugation at 5000 rpm at 4 °C for 10 min. The supernatant was transferred to a new 1.5 mL tube and centrifugated at 15,000 rpm at 4 °C for another 30 min. Immunoprecipitation was performed using the supernatant of the whole-cell extract (WCE) by rotating end-over-end at 4 °C overnight with antibody-conjugated Sepharose beads. Specifically, 0.5 µg monoclonal anti-FLAG M2 antibody (F-3165, Sigma, St. Louis, MI, USA) was incubated at RT for 1 h with recombinant protein G Sepharose 4B beads (101243, Thermo Fisher, Waltham, MA, USA) pre-blocked with 10% BSA/PBST. The beads were pelleted and washed three times with PBS, and bound protein was eluted with 1 mg/mL 3× FLAG peptide by shaking in a cold room for 1 h. The eluate was kept on ice or at −80 °C.

### 2.5. SDS-PAGE Analysis and Western Blots

The immunoprecipitation samples were analyzed with SDS-PAGE and Western blots. Firstly, the eluate was boiled in 2× protein loading dye for 10 min, then cooled down on ice. After running the SDS-PAGE, the gels were stained with Coomassie blue for 20 min, followed by destaining for 1 h. For Western blots, N protein and negative samples were blocked with 5% milk in PBS containing 0.1% Tween-20 (PBST), and then blotted with monoclonal α-FLAG M2 antibody (F-3165, Sigma) diluted 1:2000 in 5% BSA/PBST. Then, the blots were incubated with goat anti-mouse IgG (H + L) HRP-linked secondary antibody ((32430, Thermo Fisher)) diluted 1:10,000 in blocking buffer. Blots were visualized with the BioRad ChemiDoc Touch Imaging system.

### 2.6. Mass Spectrometry Sample Preparation

For the mass spectrometry samples, we ran immunoprecipitation (IP) samples in the SDS-PAGE system at only 0.5–1 cm using separate gel, and then stained the samples with Coomassie blue. They were then destained overnight (Figure S2). The gel band corresponding to the size of the SARS-CoV-2 N protein was cut out from the gel and named as N-only; the rest of the gel was named as N pull-down since N-associated proteins might have different molecular weights. All the samples, as well as the negative control (Mock), were treated in the same way using in-gel digestion. We diced each gel slice into 1 mm^2^ and added 500 µL of 25% acetonitrile (ACN)/50 mM ammonium bicarbonate (ABC) for 10 min. The samples were sonicated for 15 min; then, the solution was discarded and replaced with 500 µL of 50% ACN/50 mM ABC. After repeating the above step twice, the samples were sonicated again for 15 min. Then, the solution was discarded and replaced with 500 µL of 100% ACN. The samples were sonicated for 10 min; then, the solution was dried using a speed-vac. The samples were reduced with the addition of 400 µL of 10 mM Dithiothreitol (DTT)/50 mM ABC and incubated at 37 °C for 1 h, after which 49 µL of 500 mM Iodoacetamide (IAA) was added. The samples were incubated in the dark at room temperature for 30 min. The samples were washed twice with 500 µL of 100% ACN with 5 min sonication, then dried using a speed-vac. The samples were then suspended in 100 µL of trypsin solution and incubated at 37 °C for 16 h. Then, 500 µL of 25% ACN/5% Hydrogen acetate (HAc) was added to the trypsin-digested samples and the samples were sonicated for 20 min. An additional 300 µL of 50% ACN/5% HAc was added to the samples and the samples were sonicated for 20 min. The solution was then dried using a speed-vac and desalted using a C18 zip-tip (Waters, Milford, MA, USA). The peptide solution was then dried with a speed-vac and stored at −80 °C until LC-MS/MS analysis.

### 2.7. Mass Spectrometry Running and Data Analysis

For the N immunoprecipitation samples, liquid chromatography was performed on a Waters nanoAcquity UPLC in single-pump trapping mode with a Thermo PepMap RSLC C18 EASY-spray column (2 μm, 100 Å, 75 μm × 25 cm) and a Waters Symmetry C18 trap column (5 μm, 100 Å, 180 μm × 20 mm). The solvents used were as follows: A: water with 0.1% formic acid; B: acetonitrile with 0.1% formic acid. The samples were separated at 300 nL/min with a 260 min gradient starting at 3% B and increasing to 30% B from 1 to 230 min, then to 85% B at 240 min. They were held for 10 min, then back to 3% B in 10 min. Mass spectrometry data was acquired using a Thermo Orbitrap Fusion in data-dependent mode. A full scan was conducted using the Orbitrap at 60 k resolution in positive mode. Precursors for MS2 were filtered by monoisotopic peak determination for peptides (this was set to small molecule for the analysis), an intensity threshold of 5.0 × 10^3^, a charge state of 2-7, and 60 s of dynamic exclusion after 1 analysis with a mass tolerance of 10 ppm. Collisionally induced dissociation spectra were collected in MS2 at 35% energy and an isolation window of 1.6 *m*/*z*. The raw data were processed and analyzed using MaxQuant (version 2.0.3.1) against a custom FASTA database with the SARS-CoV-2 N sequence for the N protein sequence. The precursor mass tolerance was set to 10 ppm and the fragment mass tolerance to 0.6 Da. The results were filtered to a strict 1% false discovery rate.

### 2.8. Co-Immunoprecipitation (Co-IP)

Next, 6 × 10^5^ 293T cells were plated in each well of a 6-well tissue culture plate the day before transfection. They were cotransfected with 0.5 µg each of pCAGGS-encoding Myc-hG3BP1/2 and SARS-CoV-2 N-FLAG, or their mutants using PEI. At 48 h post-transfection, cells were collected for lysis in NP-40 buffer (1% NP-40, 50 mM Tris (pH 7.4), 150 mM NaCl, 10% glycerol, and 1 mM DTT) supplemented with 1 mM PMSF. The lysis procedure was performed by rotating end-over-end at 4 °C for 30 min, followed by centrifugation at 15,000 rpm at 4 °C for 15 min. Co-IP was performed using the supernatant of the WCE by rotating end-over-end at RT for 1 h with antibody-conjugated Sepharose beads. Specifically, 0.5 µg of monoclonal anti-FLAG M2 antibody (F3165, Sigma) was incubated at RT for 1 h with recombinant protein G Sepharose 4B beads (101243, Thermo Fisher) pre-blocked with 10% BSA/PBST. The beads were pelleted and washed three times with PBS, and bound protein was eluted through boiling in 2× protein loading buffer for 10 min. Co-IPs and WCE were analyzed by SDS–PAGE followed by immunoblotting with mouse anti-Myc (9B11, Cell Signaling) and anti-FLAG primary antibodies followed by goat anti-mouse IgG (H + L) HRP-linked secondary antibodies (32430, Thermo Fisher). SDS–PAGE was also immunoblotted with HRP-conjugated GAPDH monoclonal antibodies (HRP-60004, Proteintech, Rosemont, IL, USA).

### 2.9. Immunofluorescence Microscopy

To analyze the colocalization of over-expressed SARS 2 N protein and hG3BP1 or mutants, 293T cells grown on glass coverslips were cotransfected using PEI with 0.5 µg each of pCAGGS-encoding SARS-CoV-2-N-FLAG and Myc-hG3BP1, or their mutants. At about 30 h post-transfection, cells were stressed with 500 µM sodium arsenite for 60 min. After the treatment, the cells were fixed in 4% paraformaldehyde for 15 min at 4 °C, and then blocked with 2% BSA/PBST at RT for 2 h. The cells were incubated with mouse anti-Myc and rabbit anti-FLAG primary antibodies (catalog no. 14793S, Cell Signaling, Danvers, MA, USA) for 2 h at 30 °C, followed by three 5 min washes with PBS. Alexa-Fluor-488-conjugated anti-mouse and Alexa-Fluor-555-conjugated anti-rabbit secondary antibodies and DAPI were added to the cells for 1 h at 30 °C. After three additional washes with PBS, the coverslips were mounted onto slides with Vectashield (Vector Laboratories, Newark, CA, USA). Images were captured using a Zeiss LSM 880 confocal microscope (Zeiss, Oberkochen, Germany) equipped with a ×63/1.4 oil objective lens. Images were collected at 16 bits and at a resolution of 1024 × 1024 pixels. Image processing and analysis were carried out using ZEN 3.4 software.

### 2.10. Statistical Analysis

Two-tailed *t*-tests were used for Figures 4B and 5B. Figure 4B shows the cell percentage with SG formation with and without the co-localization of N protein. Data are mean ± SEM. Figure 5B shows the multistep growth curve of SARS-CoV-2 virus in different cells at an MOI of 1.0. Data are mean ± SEM (ns, *p* > 0.05; *, *p* ≤ 0.1; **, *p* ≤ 0.01; ***, *p* ≤ 0.001, ****, *p* ≤ 0.0001).

## 3. Results

### 3.1. Identification of Host Factors Associated with SARS-CoV-2 N Protein Through Affinity-Purification-Based Liquid Chromatography–Mass Spectrometry (LC-MS)

**Figure 1:**
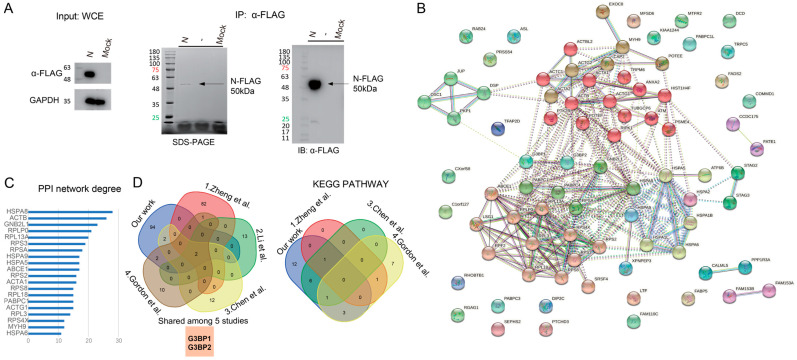
Host factors associated with SARS-CoV-2 N protein. (**A**) Expression of Flag-tagged SARS-CoV-2 N protein. Human 293T cells were transfected with a plasmid encoding a SARS-CoV-2 N-FLAG construct. At 48 h post-transfection, cells were harvested and protein extracts were subjected to immunoprecipitation using an anti-Flag antibody and then analyzed by SDS-PAGE and immunoblotting (Mock: non-transfected cells; N: cells transfected with the N-FLAG plasmid, Coomassie blue staining (middle panel), and Western blot analysis (left and right panels)). (**B**) Network of SARS-CoV-2 N protein interacting proteins generated with STRING. (**C**) Top twenty hub proteins in PPI network. (**D**) Venn diagram showing the overlapping N protein interactions and overlapping KEGG pathway between our study and data obtained from four references [23,24,25,26].

To investigate the host protein–protein interactions involving SARS-CoV-2 N protein, we applied a strategy that involved the generation of a recombinant N protein tagged with a C-terminal FLAG epitope. Given the published structure of the N protein [5,17], we reasoned that this modification would not disrupt its native conformation, thereby preserving the authentic protein structure essential for cellular and biochemical analyses. To test its expression, we transiently overexpressed the FLAG-tagged N protein in 293T cells and subjected the cell lysates (Figure 1A left) to affinity immunoprecipitation to isolate the tagged protein. Immunoblot analysis of the purified protein revealed a robust expression of SARS-CoV-2 N-FLAG fusion protein in 293T cells (Figure 1A). Furthermore, Coomassie blue staining (Figure 1A middle) and Western blot analysis (Figure 1A right) confirmed the specificity of the purification, demonstrating the selective enrichment of SARS-CoV-2 N protein (Figure 1A).

To elucidate the host proteins that interact with SARS-CoV-2 N protein, we performed a liquid chromatography–mass spectrometry (LC-MS) approach to analyze the proteins co-purified with the N-Flag fusion protein. Our analysis revealed a total of 201 proteins in 293T cells transfected with the N-FLAG construct (Table S1-1). However, to minimize the likelihood of false positives, we compared the intensity of each protein in three samples: Mock, N only, and N pull-down. We considered only those proteins that exhibited an intensity ratio of ≥1:1 between the N-only or N pull-down sample and the Mock sample to be authentic interactions. This filtering step yielded a dataset of 100 unique, high-confidence SARS-CoV-2 N-interacting proteins (Table S1-2). The fact that we still detected a substantial number of host proteins associated with the SARS-CoV-2 N protein, even after stringent filtering, is in line with the multifunctional and central role of the N protein in the viral life cycle. Nevertheless, it is worthwhile noting that even with stringent filtering, the non-specific association of highly abundant cellular proteins like HSP and ACTB remains a common consideration in IP-based analyses. To visualize these interactions, we constructed a protein–protein interaction (PPI) network, which comprised 82 nodes and 262 edges (Figure 1B). Notably, the top ten hub proteins, as defined by their degree of connectivity, were HSPA8, ACTB, GNB2L1, RPLP0, RPL13A, RPS3, RPSA, HSPA9, HSPA5, and ABCE1 (Figure 1C). Furthermore, to validate our LC-MS analysis, we mapped the identified peptides onto the SARS-CoV-2 N protein (Figure S1). Our analysis revealed that 76.2% of the N protein’s amino acid sequence was covered by the identified peptides, thereby confirming the reliability of our LC-MS analysis.

To further determine the key host factors associated with SARS-CoV-2 N protein, we reconciled and integrated divergent SARS-CoV-2 OMICs studies with our own [23,24,25,26]. Notably, a striking feature of this analysis was the paucity of overlap between the interaction partners identified in these studies. However, a common thread emerged among the interacting proteins, with the stress granule (SG) assembly factors G3BP1 and G3BP2 being the sole shared interactors among the different studies (Figure 1D and Table S2-1). Furthermore, we observed that eight proteins in our PPI network had direct interactions with G3BP1 or G3BP2, including GNB2L1, PABPC1, PABPC4, PKP1, ABCE1, ACTB, HSPA8, and HSPA9. Intriguingly, six of these proteins were among the top twenty hub proteins in our PPI network, suggesting a critical role for these proteins in the SARS-CoV-2 N protein interactome. To gain further insights into the functional significance of these interactions, we performed a Kyoto Encyclopedia of Genes and Genomes (KEGG) pathway analysis on our data and the references. Notably, each reference shared at least one pathway with our study, including pathways related to protein processing in the endoplasmic reticulum (hsa04141) and prion disease (hsa05020) (Table S2-2).

**Figure 2:**
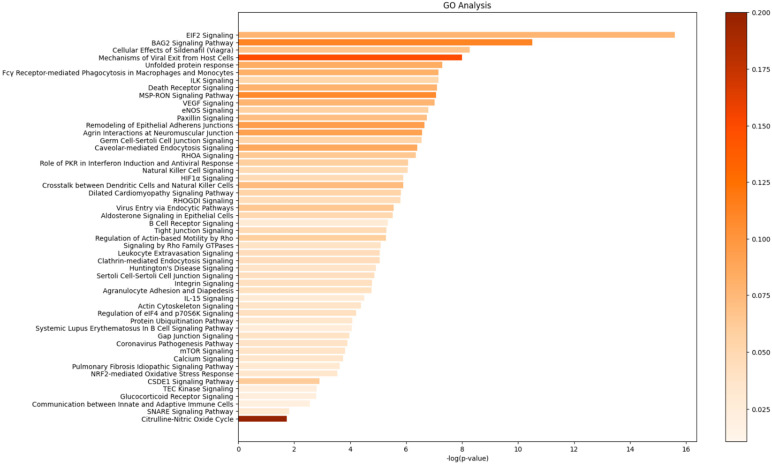
Top fifty biological processes enriched in N protein interacting proteins based on gene ontology analysis.

To gain a further understanding of the complex interactions between SARS-CoV-2 N protein and host cellular factors, we performed a comprehensive pathway analysis using QIAGEN Ingenuity Pathway Analysis. This approach revealed the top fifty-two biological processes associated with N protein interactions, as illustrated in Figure 2. Notably, the results highlighted a pronounced enrichment for SG assembly, unfolded protein response, and the role of PKR in IFN and antiviral response. Specifically, several members of the heat shock protein (HSP) family, including HSPA1A/HSPA1B, HSPA1L, HSPA2, HSPA5, HSPA6, HSPA8, and HSPA9, were implicated in these pathways (Table S3-1). Furthermore, our analysis identified EIF2 signaling as a significantly enriched pathway within the N protein interactome. We also observed an association between N protein interactions and various defense mechanisms. To further elucidate the functions of G3BP1 and G3BP2, we performed a network analysis, which revealed distinct interactions for each protein. G3BP2 was found to participate in a network related to cellular compromise, inflammatory response, and protein synthesis (Network 1, Table S3-2), whereas G3BP1 was associated with cellular function and maintenance, humoral immune response, and inflammatory response (Network 2, Table S3-2). Despite the established roles of G3BP1 and G3BP2 in innate immune response and protein synthesis, as well as their essential involvement in SG formation [27,28,29], recent studies have revealed a paradoxical effect of these proteins on SARS-CoV-2 infection, with some reports suggesting a conflicting impact on viral replication [15,18,21,30]. To resolve this apparent discrepancy, we undertook a direct comparison of live SARS-CoV-2 infection in cells either expressing or lacking these two cellular proteins.

### 3.2. Molecular Mechanism of Association Between SARS-CoV-2 N Protein and GBBP1 or G3BP2

To explore the molecular mechanisms that underlie the function of G3BP1/2 in SARS-CoV-2 infection, we first performed a detailed investigation of the interactions between G3BP1/2 and SARS-CoV-2 N protein. The crystal structure of SARS-CoV-2 N_1-25_ and the G3BP_NTF2_ complex [17] revealed a high degree of surface complementarity between residues 12-22 of SARS-CoV-2 N protein and the surface groove of G3BP1/2_NTF2_. We exploited G3BP1 protein as a paradigmatic representative in our following evaluation of the critical residues requisite for interaction.

**Figure 3:**
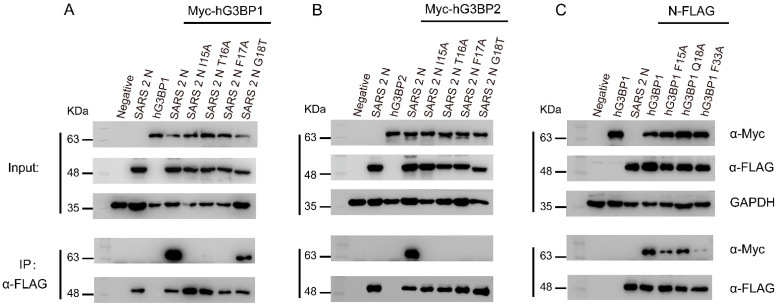
Critical residues required for SARS-CoV-2 N-G3BP1/2 interaction. (**A**) Co-IP analysis showing the effects of SARS-CoV-2 N protein mutations on N-G3BP1 interaction. (**B**) Co-IP analysis showing the effects of SARS-CoV-2 N protein mutations on N-G3BP2 interaction. (**C**) Co-IP analysis showing the effects of hG3BP1 protein mutations on G3BP1–SARS-CoV-2 N interaction. Immunoblot analysis of 293T cells transfected with indicated plasmids encoding Myc-tagged G3BP1/G3BP2, and SARS-CoV-2 N-FLAG using antibodies against Myc, Flag, and GAPDH. The experiments were performed three times with consistent results.

Also guided by structural information [17], we introduced site-specific amino acid substitutions into SARS-CoV-2 N protein, including I15A, T16A, F17A, and G18T, and into the G3BP1 protein, including F15A, Q18A, and F33A. The effects of these mutations on SARS-CoV-2 N-hG3BP1/2 interaction were assessed by co-immunoprecipitation (Co-IP) and immunofluorescence (IF) analysis using 293T cells transfected with Myc-tagged hG3BP1 (Myc-hG3BP1) and FLAG-tagged SARS-CoV-2 N protein (SARS-CoV-2 N-FLAG). The results showed that wild-type (WT) SARS-CoV-2 N protein interacted strongly with hG3BP1 (Figure 3A) and hG3BP2 (Figure 3B), confirming their interaction in human cells. However, the introduction of mutations into SARS-CoV-2 N protein resulted in a significant decrease in the level of hG3BP1 co-precipitated (Figure 3A), and the co-precipitation of hG3BP2 became undetectable with any of the N protein mutants (Figure 3B), indicating that these sites are critical for SARS-CoV-2 N-hG3BP1/2 interaction. Conversely, WT hG3BP1 protein interacted strongly with SARS-CoV-2 N protein (Figure 3C), whereas the mutations in hG3BP1 resulted in a decrease in the level of SARS-CoV-2 N-associated hG3BP1, highlighting the importance of these amino acid residues for SARS-CoV-2 N-hG3BP1 interaction.

### 3.3. The Association of SARS-CoV-2 N Protein with GBBP1 or G3BP2 Inhibits the Formation of SG

**Figure 4:**
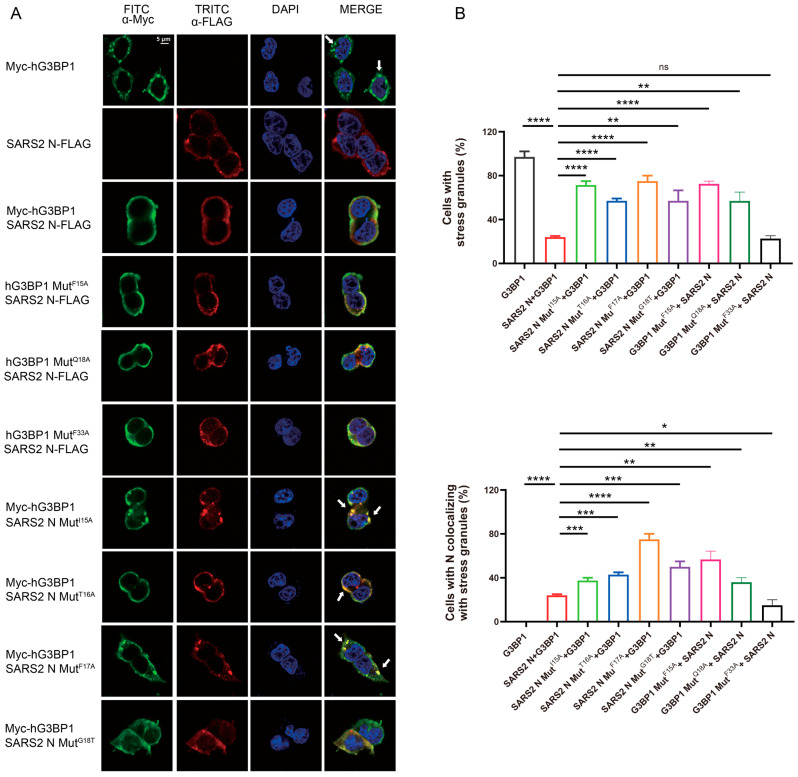
Interaction of SARS-CoV-2 N and G3BP1 suppresses SG formation. (**A**) Representative IF images of 293T cells transfected with plasmids encoding Myc-G3BP1 and/or SARS-CoV-2 N-FLAG, followed by treatment of sodium arsenite. The stress granules are marked by white arrows. (**B**) Statistical analysis of SG formation in cells and the SG colocalization of SARS-CoV-2 N protein shown in panel (**A**). Data are mean ± SEM. Statistics: two-tailed *t*-test (ns, *p* > 0.05; *, *p* ≤ 0.1; **, *p* ≤ 0.01; ***, *p* ≤ 0.001, ****, *p* ≤ 0.0001).

We next assessed the functional consequences of SARS-CoV-2 N protein and hG3BP1 interactions on SG formation. We used a sodium arsenite treatment procedure to induce SG formation in cells transfected with Myc-G3BP1 and/or SARS-CoV-2 N-FLAG [31]. Following optimization of the sodium arsenite treatment conditions, we used 500 μM and 60 min as the optimal conditions for subsequent experiments. Cells transfected with Myc-G3BP1 alone exhibited pronounced cytoplasmic puncta formation by Myc-G3BP1, indicative of SG formation (Figure 4A). In contrast, co-transfection with Myc-G3BP1 and SARS-CoV-2 N-FLAG resulted in a marked decrease in SG formation, consistent with SARS-CoV-2 N protein having a role in inhibiting SG formation. Notably, co-transfection with Myc-G3BP1 and SARS-CoV-2 N I15A or F17A mutant, or with Myc-G3BP1 and SARS-CoV-2 N T16A G18T mutant resulted in a significant restoration of SG formation, suggesting that these mutations impaired SARS-CoV-2 N protein’s ability to inhibit SG formation. Furthermore, co-transfection with SARS-CoV-2 N and Myc-G3BP1 F15A, Q18A, and F33A mutants also led to a partial restoration of SG formation, albeit to a lesser extent (Figure 4A). These findings suggest that SARS-CoV-2 N protein mutants I15A, T16A, F17A, and G18T, as well as the hG3BP1 mutants F15A and Q18A, are defective in their ability to inhibit SG formation. Interestingly, SARS-CoV-2 N-FLAG mutants I15A, T16A, F17A, and G18T, as well as the hG3BP1 mutants F15A and Q18A, were also found to be enriched in SG in these cells (Figure 4B), indicating that the partitioning of SARS-CoV-2 N protein into SG is independent of its interaction with G3BP1.

### 3.4. G3BP1 and G3BP2 Share Overlapping Roles in Suppressing SARS-CoV-2 Replication Beyond SG Pathway

**Figure 5:**
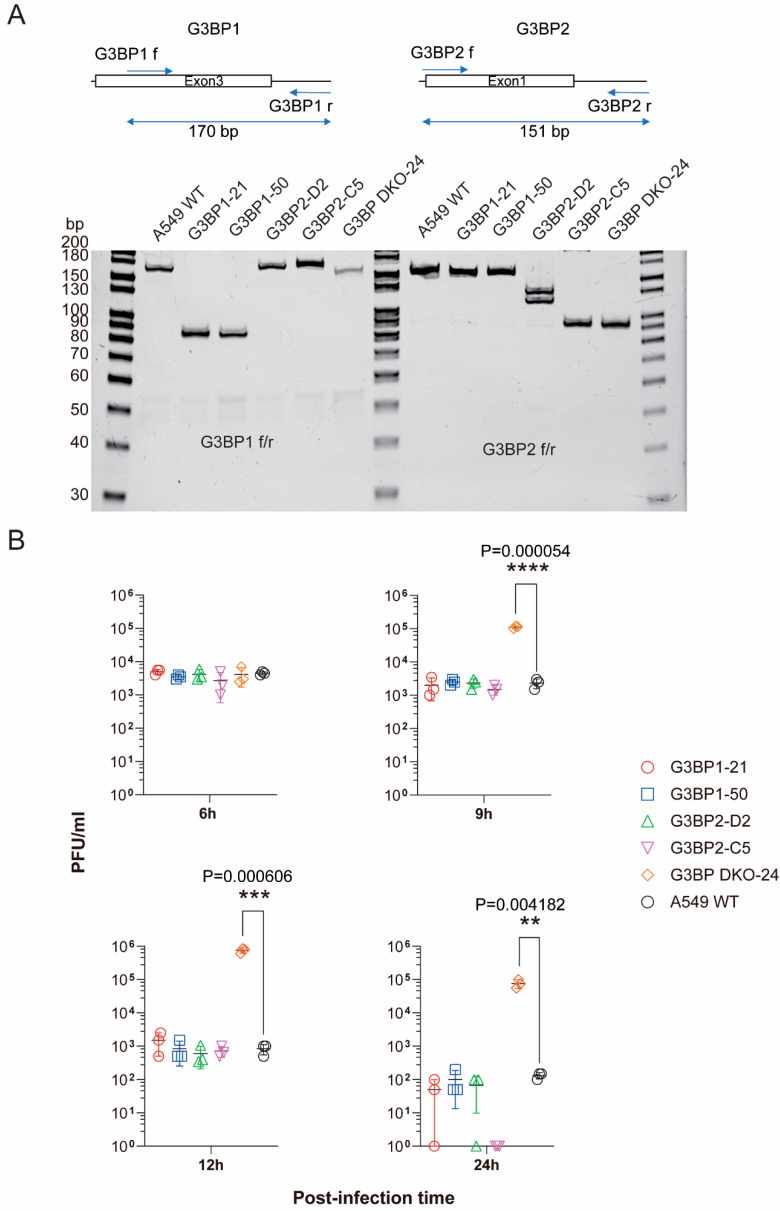
Dual ablation of G3BP1/2-enhanced SARS-CoV-2 replication. (**A**) A549 cells were engineered to carry knockout mutations in G3BP1, G3BP2, or both, using CRISPR-Cas9-mediated genome editing. To verify the specificity of the knockout mutations, PCR amplification was performed using primers flanking the targeted regions (upper panel). The resulting amplicons were then resolved by 8% PAGE gel electrophoresis (lower panel), revealing distinct size differences that corresponded to the expected knockout mutations. These PCR products were subsequently subjected to sequencing analysis to confirm the precise nature of the genetic alterations in the G3BP1 and G3BP2 genes. (**B**) Viral titers were measured at designated time points after infection with SARS-CoV-2 at a multiplicity of infection (MOI) of 1.0. PFU: Plaque-forming unit. (**, *p* ≤ 0.01; ***, *p* ≤ 0.001, ****, *p* ≤ 0.0001).

Despite the substantial body of evidence accumulated from earlier studies and other investigations [15,18,21,30], the roles of G3BP1 and G3BP2 in SARS-CoV-2 replication remain controversial. To determine the functions of these host proteins in the context of SARS-CoV-2 replication, we performed a comprehensive analysis of live virus replication in cells either devoid of or expressing G3BP1 and G3BP2. Initially, we generated A549 cells with targeted deletions of G3BP1 or G3BP2 through CRISPR-Cas9, respectively. We isolated two independent G3BP1-deficient cell lines (G3BP1-21 and G3BP1-50) and two G3BP2-deficient cell lines (G3BP2-D2 and G3BP2-C5), each carrying distinct mutations that introduced premature stop codons in exon 5, exon 3, and exon 2, respectively (Figure S3). Unexpectedly, virus replication in these mutant cells was found to be comparable to that of WT A549 cells. Likely due to the low expression of hACE2 in our A549 cells, the virus replication is low, as shown by wt SARS-CoV-2 titers being maintained around 10^3^ to 10^4^ PFU/mL until 12 h post-infection. Given that SARS-CoV-2 N protein is capable of inhibiting the SG pathway through interactions with G3BP1/2, these findings suggest that the virus has evolved a mechanism to circumvent the SG pathway, likely mediated by its N protein–G3BP1/2 interaction. To further test whether G3BP1/2 proteins play additional roles beyond the SG pathway impacting on SARS-CoV2 replication, we generated a G3BP double knockout cell line (G3BP DKO-24), which carries premature stop codons in G3BP2 exon 2 and G3BP1 exon 3 (Figure 5A). Intriguingly, SARS-CoV-2 replication was significantly enhanced in these cells lacking both G3BP1 and G3BP2 compared to WT cells. This observation implies that G3BP1 and G3BP2 proteins exhibit overlapping antiviral functions against SARS-CoV-2, distinct from their roles in the SG pathway (Figure 5). Moreover, our findings suggest that the virus has not yet developed an effective countermeasure to antagonize this antiviral activity.

In summary, our study has identified that G3BP1 and G3BP2 proteins are key factors associated with SARS-CoV-2 N protein. Notably, our findings indicate that the simultaneous depletion of both G3BP1/2 proteins is required to effectively enhance viral replication, thereby highlighting the overlapping antiviral role shared by these two cellular proteins. We further identified the molecular mechanisms required for their interaction and shed light on one functional consequence of the interaction. These results have significant implications for the development of novel host-directed antiviral strategies against SARS-CoV-2.

## 4. Discussion

The COVID-19 pandemic has underscored the need for a comprehensive understanding of SARS-CoV-2 biology, and the N protein is a crucial component in this regard. As the most abundant viral protein produced during viral infection, N protein plays a central role in viral replication, transcription, and translation, as well as in the evasion of host immune responses. Furthermore, its interaction with host cellular factors, including G3BPs involved in SG formation, highlights the N protein’s critical function in manipulating cellular machinery to favor viral replication. Elucidating the molecular mechanisms governing SARS-CoV-2 N protein’s interactions with host factors is essential to uncover potential targets for therapeutic intervention and develop effective countermeasures against SARS-CoV-2. As new variants of SARS-CoV-2 continue to emerge, a detailed understanding of N protein’s functions and interactions will inform strategies for controlling and mitigating the impact of future SARS-CoV-2 variants or coronavirus pandemics.

Like other RNA viruses, SARS-CoV-2 relies heavily on exploiting host cellular machinery to establish infection, primarily through the interactions between viral proteins and host factors. Various large-scale mass spectrometry-based screens have identified numerous host factors that interact with SARS-CoV-2 N protein. Notably, our study and others consistently identified G3BP1 and G3BP2 as key host proteins that interact with SARS-CoV-2 N protein (Figure 1D) [23,24,25,26]. These findings suggest that G3BP1 and G3BP2 are essential host factors that enable N protein to modulate the cellular environment in favor of viral infection. Furthermore, a comparative analysis of KEGG pathways potentially perturbed by the N protein revealed that stress responses are among the top affected pathways (Figure 1D) [23,24,25,26]. Collectively, these results indicate that SARS-CoV-2 N protein plays a critical role in hijacking host stress response pathways, particularly through the SG pathway, via its interaction with G3BP1 and G3BP2.

G3BP1 and G3BP2 are structurally homologous proteins. Both possess a conserved domain architecture comprising five distinct motifs (starting from their N-terminal ends): the nuclear transport factor 2 (NTF2) domain, an acidic-rich region, a proline-rich (PxxP) motif, an RNA recognition motif (RRM), and an RGG domain (arginine–glycine-rich boxes) [32,33,34]. Both proteins play pivotal roles in the SG pathway. G3BP1 plays a crucial role in initiating the formation of SGs, as demonstrated by the fact that its knockdown significantly reduces SG assembly [29,35]. G3BP2, a close relative of G3BP1, shares a similar domain architecture [36]. Notably, the overexpression of G3BP2, like G3BP1, can induce SG formation even in the absence of stress stimuli [29]. Furthermore, G3BP2 and G3BP1 can form homo- or heterodimers, which are essential for SG formation [37]. The expression levels of G3BP1 have been shown to correlate with the number of early SGs, and the overexpression of G3BP1 enhances SG formation [38,39]. SGs are dynamic, cytoplasmic foci that form through the aggregation of untranslated messenger ribonucleoproteins (mRNPs), which accumulate as a result of stress-induced translation arrest [40]. These transient, non-membrane-bound organelles serve as hubs that coordinate stress responses and cell fate decisions via sequestering key host factors required in translation [41,42].

Given the central role of SGs in halting protein synthesis, it is reasonable for viruses to develop diverse strategies to hijack SGs to their advantage by manipulating G3BPs through interaction, degradation, or modification. Depending on their optimal infection strategy, viruses can either enhance or inhibit the formation of SGs. For example, the HIV Gag protein interacts with G3BP1 to suppress the formation of SGs, thereby establishing productive infection [43]. On the other hand, Vaccinia virus has been found to recruit G3BP to facilitate its replication at different stages of the viral life cycle [44]. During the early stages of infection, the viral factory is surrounded by the rough endoplasmic reticulum, which attracts essential translation initiation factors, including G3BP1 and Caprin-1 [44]. The seemingly contradictory effects of G3BP on viral infection are likely attributed to the intricate composition and diverse substrates of SGs, which can influence viral replication in complex and multifaceted ways.

Intriguingly, existing studies on SARS-CoV-2 have reported directly conflicting results on the effect of G3BP modulation on viral replication. On one hand, some research has found that inhibiting or depleting G3BP1 protein actually enhances viral replication [15,18], whereas others have observed a completely opposite effect [21,30].

Therefore, elucidating the roles of G3BP1 and G3BP2 is crucial, particularly in light of the contradictory reports suggesting their complex effects on SARS-CoV-2 infection [15,18,21,30]. To resolve this ambiguity, we took a direct approach by generating A549 cell lines deficient in G3BP1, G3BP2, or both, though CRISPR, and then examined SARS-CoV-2 replication in these cells compared to WT A549. Intriguingly, our experiments revealed that the replication kinetics of SARS-CoV-2 in cells deficient in either G3BP1 or G3BP2 were indistinguishable from those in WT A549 cells. This finding provides compelling evidence for the notion that G3BP1 and G3BP2 play redundant roles in the activation of SG pathways, thereby underscoring the inherent plasticity of this cellular response mechanism. Notably, our results showed that the deletion of both G3BP1 and G3BP2 results in a significantly increased SARS-CoV-2 replication in cells lacking both host factors compared to control A549 cells (Figure 5B). This unarguably highlights the importance of G3BP1 and G3BP2 as suppressive host factors during SARS-CoV-2 infection. This finding implicates that G3BP1 and G3BP2 share overlapping functions that extend beyond the canonical role of SG pathways, possibly subverting additional aspects of the viral replicative cycle, and underscoring the intricate web of host–viral interactions that govern the progression of infection, warranting further investigation.

Additionally, our results have further elucidated the interaction between G3BP1 and the SARS-CoV-2 N protein, revealing new details about this critical interface. In addition to the previously reported F17A residue [8], our results identified additional residues that are required for the interaction between G3BP1 and N protein (Figure 3). This finding revealed a more complex binding interface that can be targeted to disrupt viral replication. Furthermore, these observations highlighted the need to further investigate the impact of these residues on viral replication. Next, we would use our established reverse genetics system for SARS-CoV-2 to generate recombinant viruses harboring individual or combinatorial mutations of these residues and compare their replication kinetics to those of the wild-type virus.

In conclusion, our study highlights the complex interplay between host factors and SARS-CoV-2 pathogenesis, emphasizing the need for rigorous experimental design to elucidate the roles of these factors in viral infection. Our findings have significantly deepened our understanding of the interactions between SARS-CoV-2 N protein and G3BP1/2, elucidating the consequences of this interplay on viral infection. Notably, our results collectively point to SARS-CoV-2 N protein as a promising target for antiviral therapy, warranting further investigation into potential antivirals targeting the interaction between N and G3BP1/2 proteins.

## Data Availability

The authors declare that the data supporting the findings of this study are available within the paper.

## Supplementary Materials

The following supporting information can be downloaded at: https://www.mdpi.com/article/10.3390/v17070912/s1, Figure S1: Linear representation of the SARS-CoV-2 nucleocapsid (N) protein, highlighting peptides identified by LC-MS analysis. The full-length protein sequence is shown, with tryptic digestion sites indicated in green. Red indicates the peptides that were successfully identified in our LC-MS analysis; Figure S2: Coomassie blue-stained protein gel of pull-down assays using SARS-CoV-2 N protein versus mock samples, prepared for LC-MS analysis to compare interacting proteins; Figure S3: Schematic of specific mutations in genes induced by CRISPR-Cas9 in A549 Cell Lines; Table S1-1: The SARS-CoV-2 N protein associated host proteins obtained by MS data search; Table S1-2: The selected SARS-CoV-2 N associated host proteins after intensity comparison; Table S2-1: The Protein IDs summary and Venn results from our analysis and previously studies; Table S2-2: The KEGG pathway summary and Venn results from our analysis and previously studies; Table S3-1: The result of QIAGEN Ingenuity Pathway Analysis: Table S3-2: The network analysis from QIAGEN Ingenuity Pathway Analysis.
