# Supplementary material for: Beyond Stress Granules: G3BP1 and G3BP2 Redundantly Suppress SARS-CoV-2 Infection
Source: Viruses. 2025 Jun 27;17(7):912. doi: 10.3390/v17070912 (PMC12300500; doi:10.3390/v17070912)
Supplement: Supplementary file 1 [file viruses-17-00912-s001.zip › viruses-3225000-supplementary.pdf]

Supplementary Material

Beyond Stress Granules: G3BP1 and G3BP2 Redundantly Suppress SARS-CoV-2 Infection

Duo Xu *et al.*

*Corresponding author: Rong Hai, ronghai@ucr.edu.

Figure supplement:

This PDF file includes:

Figure S1.

Figure S2.

Figure S3.

The description of supplementary tables.

MSDNGPQNQRNAPRITFGGPSDSTGSNQNGERSGARSKQRRPQGLPNNTASW

FTALTQHGKEDLKFPRGQGVPINTNSSPDDQIGYYRRATRRIRGGDGKMKDLSPR

WYFYYLGTGPEAGLPYGANKDGIIWVATEGALNTPKDHIGTRNPANNAAIVLQLP

QGTTLPKGFYAEGSRGGSQASSRSSSRSRNSSRNSTPGSSRGTSPARMAGNGGD

AALALLLLDRLNQLESKMSGKGQQQQGQTVTKKSAAEASKKPRQKRTATKAYNV

TQAFGRRGPEQTQGNFGDQELIRQGTDYKHWPQIAQFAPSASAFFGMSRIGME

VTPSGTWLTYTGAIKLDDKDPNFKDQVILLNKHIDAYKTFPPTEPKKDKKKADET

QALPQRQKKQQTVTLLPAADLDDFSKQLQQSMSSADSTQA

Figure S1: Linear representation of the SARS-CoV-2 nucleocapsid (N) protein, highlighting peptides identified by LC-MS analysis. The full-length protein sequence is shown, with tryptic digestion sites indicated in green. Red indicates the peptides that were successfully identified in our LC-MS analysis.

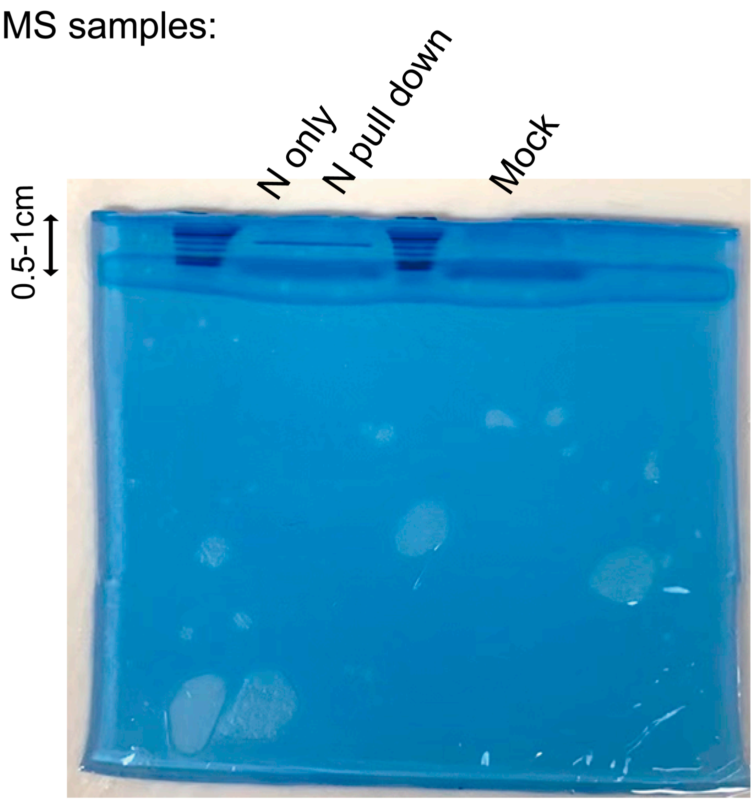

Figure S2: Coomassie blue-stained protein gel of pull-down assays using SARS-CoV-2 N protein versus mock samples, prepared for LC-MS analysis to compare interacting proteins.

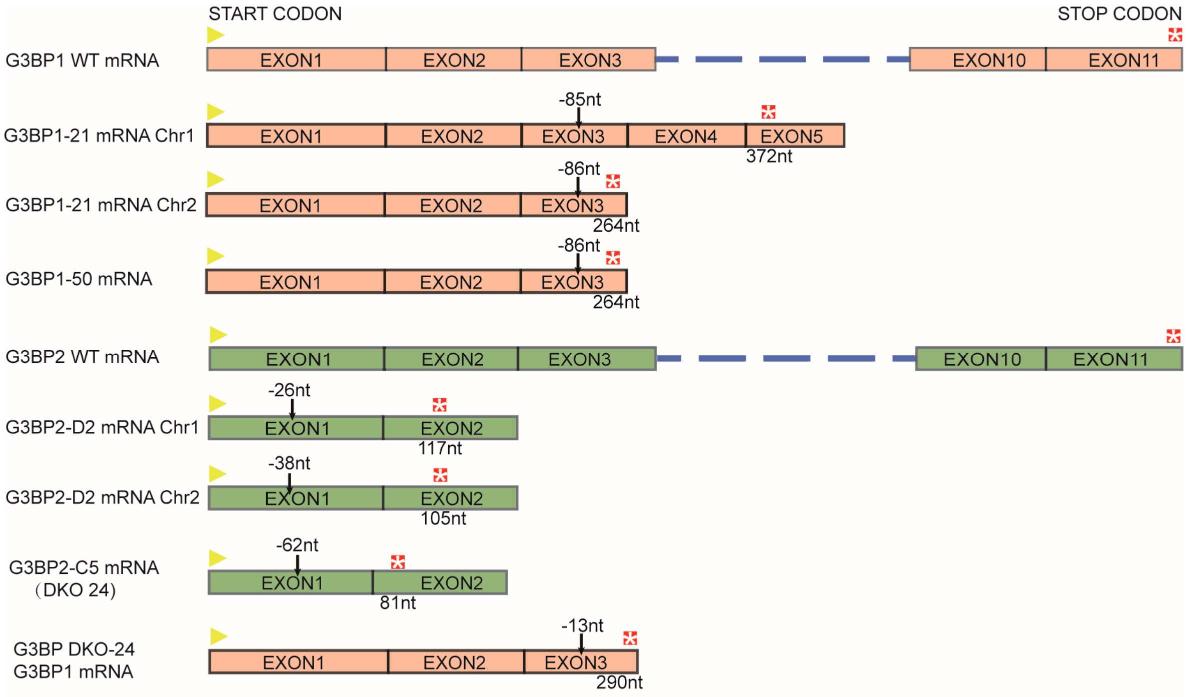

Figure S3: Schematic of specific mutations in genes induced by CRISPR-Cas9 in A549 Cell Lines

Table S1-1
The SARS-CoV-2 N protein associated host proteins obtained by MS data search.

Table S1-2
The selected SARS-CoV-2 N associated host proteins after intensity comparison.

Table S2-1
The Protein IDs summary and Venn results from our analysis and previously studies.

Table S2-2
The KEGG pathway summary and Venn results from our analysis and previously studies.

Table S3-1
The result of QIAGEN Ingenuity Pathway Analysis.

Table S3-2
The network analysis from QIAGEN Ingenuity Pathway Analysis.
